# Supplementary material for: Preparation of Novel Mesoporous LaFeO3-SBA-15-CTA Support for Syngas Formation of Dry Reforming
Source: Nanomaterials (Basel). 2022 Apr 24;12(9):1451. doi: 10.3390/nano12091451 (PMC9105762; doi:10.3390/nano12091451)
Supplement: Supplementary file 1 [file nanomaterials-12-01451-s001.zip › Supporting information-revision-1.pdf]

Preparation of Novel Mesoporous $LaFeO_3$-SBA-15-CTA Support for Syngas Formation of Dry Reforming

Luming Li [1,2,3], Song Wu [1], Hongmei Li [1], Jie Deng [1,*] and Junshan Li [2,*]

1. College of Food and Biological Engineering, Chengdu University, Chengdu 610106, China; liluming@cdu.edu.cn (L.M.); wusong@stu.cdu.edu.cn (S.W.); lihongmei@cdu.edu.cn (H.M.); dengjie@cdu.edu.cn (J).
2. Institute for Advanced Study, Chengdu University, Chengdu 610106, China; liluming@cdu.edu.cn (L.M.); lijunshan@cdu.edu.cn (J.S.).
3. Department of Chemical Engineering, Sichuan University, Chengdu 610065, China; liluming@cdu.edu.cn (L.M.).

* Correspondence: dengjie@cdu.edu.cn (J); lijunshan@cdu.edu.cn (J.S.).

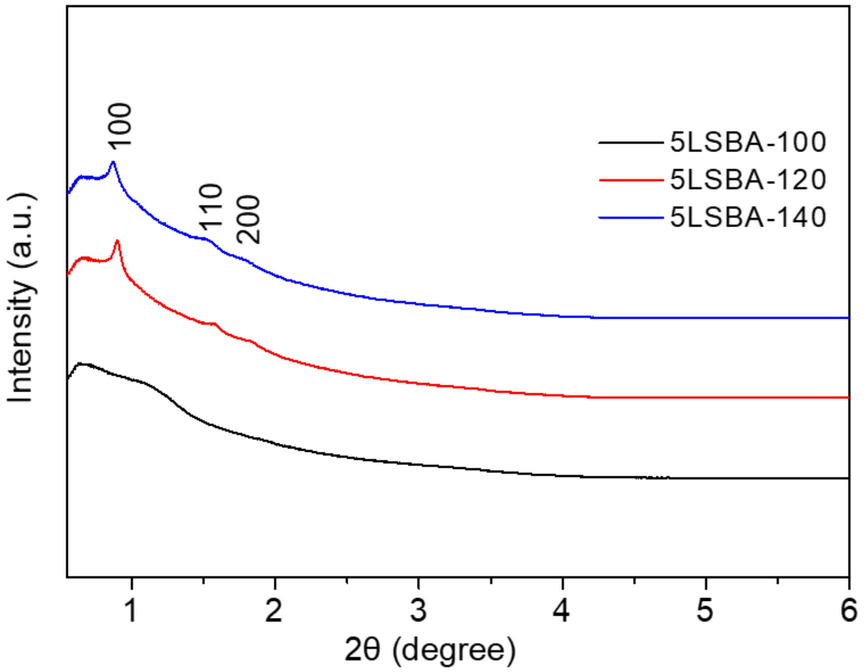

Figure S1. Small-angle XRD patterns of the $LaFeO_3$-SBA-15-CTA supports.

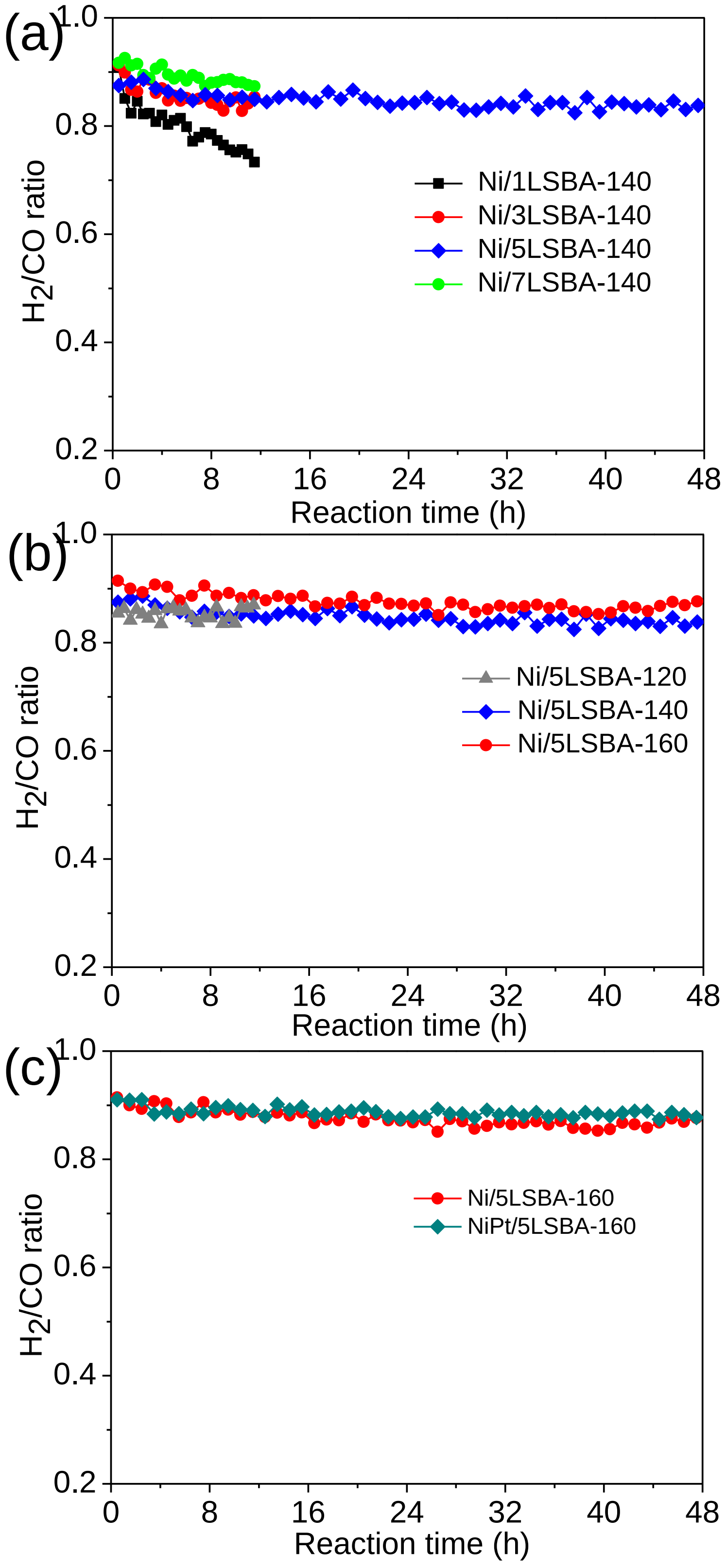

Figure S2. The enhanced catalytic performance on Ni(Pt)/yLSBA-T catalysts by adjusting the $LaFeO_3$ loading (a) and hydrothermal temperature (b) as well as the Pt modification (c). (GHSV=20,000 mL·g_{cat}^{-1}·h^{-1}).

Table S1. Preparation conditions and grain sizes of the synthesized catalysts.

**Sample name.**	**$LaFeO_3$ content (wt.%)**	**Hydrothermal temperature (°C)**	**Particle size of NiO (nm)**
NiO/3LSBA-140	30	140	18.2
NiO/5LSBA-140	50	140	14.3
NiO/7LSBA-140	70	140	12.8
NiO/5LSBA-120	50	120	15.3
NiO/5LSBA-160	50	160	13.2
$NiOPtO_x$/5LSBA-160	50	160	13.0
